# Cleavage-stage embryo transfer: we’ll never let it go

**DOI:** 10.1016/j.xfre.2021.06.009

**Published:** 2021-07-03

**Authors:** Michael S. Awadalla

**Affiliations:** Department of Obstetrics and Gynecology, Keck School of Medicine, University of Southern California, Los Angles, California

Improved modern embryo culture techniques such as the use of triple gas incubators and commercially available culture media have helped increase the frequency of culture to the blastocyst stage, use of preimplantation genetic testing for aneuploidy, and single embryo transfer. One could argue that cleavage-stage embryo transfer is unnecessary in modern practice. In some older patients with poor prognosis, cleavage-stage embryo transfer could even be considered futile. Results from one study indicate that the live birth rate for a single cleavage-stage embryo from a 45-year-old woman at retrieval is less than 1% (1). However, a cleavage-stage transfer is a good option for younger patients who are at risk of having no blastocyst-stage embryos to transfer based on poor embryo progression or failure to make it to the blastocyst stage in a previous cycle. Not having any embryos to transfer after culture to the blastocyst stage can be devastating. Additionally, trying a cleavage-stage transfer may help patients feel that they have tried everything they can to conceive. Some patients may want to try a transfer with autologous oocytes before moving on to using donor oocytes.

The retrospective study by Neblett et al. (2) in this issue evaluated the rates of live birth when patients with six or fewer normally fertilized two pronuclear embryos had either a fresh cleavage or blastocyst-stage transfer. The live birth rates were 25% after cleavage-stage transfers (average female age, 35.8 years) and 40% after day 5 blastocyst-stage transfers (average female age, 34.4 years). The investigators conclude that the success rate for cleavage-stage embryo transfer in patients with poor prognosis is still good enough to offer patients this option.

There are some limitations to this study. First, the comparison of the two cohorts (fresh cleavage and blastocyst-stage transfers) is not directly applicable to a specific clinical decision point. The choice patients have is to transfer at the cleavage stage or culture to the blastocyst stage (or a combination of both). With culture to the blastocyst stage, some patients would have had no blastocyst formation and would not have been included in this study. Although a recent randomized controlled trial (RCT) comparing fresh cleavage-stage transfer with fresh blastocyst-stage transfer in patients with good prognosis found that only 6 of 194 patients allocated to blastocyst transfer had no embryos to transfer (3), the rates of no blastocyst formation would be expected to be higher in patients with poor prognosis included in the study by Neblett et al. (2). Second, there is likely some residual confounding in that even after including only patients with six or fewer normally fertilized embryos, patients with good embryo growth (approximately four cells on day 2 and eight cells on day 3 of culture) were probably more likely to proceed with culture to the blastocyst stage than patients with suboptimal embryo growth. Lastly, this study compared the pregnancy outcomes of only the first (fresh) transfer. The day 5 transfer cohort had an average of 1.4 extra embryos cryopreserved compared with 0.07 embryos cryopreserved in the cleavage-stage transfer cohort. While this study provides valuable information on fresh cleavage-stage embryo transfer outcomes in patients with poor prognosis, it is unable to inform the decision of cleavage-stage embryo transfer vs. continued culture to the blastocyst stage.

An appropriately powered RCT comparing planned fresh cleavage-stage transfer with planned fresh blastocyst-stage transfer in patients with poor prognosis will likely never be conducted. The two main reasons are that the decision of cleavage or blastocyst transfer is often made while embryo culture is in progress and the sample size needed would be prohibitive. For an RCT, you would likely need three groups: transfer and freeze at the cleavage stage, transfer and freeze at the blastocyst stage, and a third group where decision-making could be made based on embryo progression. For just two groups to detect a difference in live birth rate per cycle start of 55% in one group and 45% in the other with 80% power and a two-sided type I error rate of 0.05, you would need to randomize 782 patients. This would be a difficult study to recruit patients for. To guarantee enrollment, you would likely have to offer generous financial compensation or discount some portion of the in vitro fertilization cycle costs. Assuming a compensation of $5,000 per participant, the cost would be $3,910,000 for the whole study just in patient compensation.

Unfortunately, the power analysis and study costs would be similar for any RCT evaluating the live birth rate per stimulation cycle start. This is a major reason why there are so few RCTs evaluating in vitro fertilization stimulation and embryo culture. Thankfully, RCTs for evaluating embryo transfer protocols are more feasible from a recruitment and cost standpoint.

Despite some limitations, studies such as the one by Neblett et al. (2) are helpful to inform clinical decision-making. Knowing that even patients with poor prognosis have approximately a 25% live birth rate after cleavage-stage embryo transfer is helpful for patient counseling. We cannot really prove or disprove that some patients may conceive with a cleavage-stage transfer of embryos that possibly would not have made it in culture to the blastocyst stage. Because it is impossible to show that blastocyst-stage culture is best for all patient subgroups, there will always be a role for cleavage-stage embryo transfer.
